# Migration from Epi Info to District Health Information Software 2 for Vaccine-Preventable Disease Surveillance — World Health Organization African Region, 2019–2023

**DOI:** 10.15585/mmwr.mm7323a2

**Published:** 2024-06-13

**Authors:** Oluwasegun Joel Adegoke, Audrey Rachlin, Angela Montesanti Porter, Reggis Katsande, Steve Kubenga,, Rebecca Potter, Ola Hodne Titlestad, Lucie Noubi Tchoupopnou Royd, Louie Rosencrans, Carl Kinkade,, Vittoria Crispino, Talya Shragai, Edem Kossi, Hong Anh Chu, Christopher S. Murrill, Eugene Lam, Charles S. Wiysonge,, Lawrence Kazembe, Lorenzo Pezzoli, Victor Alegana, Impouma Benido,

**Affiliations:** ^1^Global Immunization Division, Center for Global Health, CDC; ^2^Regional Office for Africa, World Health Organization, Brazzaville, Republic of the Congo; ^3^University of Oslo, Oslo, Norway; ^4^Division of Global Health Protection, Center for Global Health, CDC; ^5^Health Information System Programme, West and Central Africa, Lome, Togo; ^6^Data, Analytics and Delivery for Impact Division, World Health Organization, Geneva, Switzerland; ^7^Health Emergencies Programme, World Health Organization, Geneva, Switzerland.

**Summary**

**What is already known about this topic?**

Epi Info, a free, CDC-developed statistical software package, has limited capability to integrate with other information systems, affecting reporting timeliness and data use.

**What is added by this report?**

To facilitate access to high-quality timely epidemiologic data, the World Health Organization African Regional Office (AFRO) transitioned from Epi Info to the District Health Information Software 2 (DHIS2) platform for vaccine-preventable disease (VPD) surveillance. By February 2024, eight of 47 AFRO countries had adopted both the aggregate Integrated Disease Surveillance and Response and case-based surveillance packages, and two had successfully transferred VPD surveillance data to the AFRO regional platform.

**What are the implications for public health practice?**

Transitioning to DHIS2 supports data transmission through improved system integration and interoperability for timely detection of and response to VPD outbreaks.

## Abstract

High-quality vaccine-preventable disease (VPD) surveillance data are critical for timely outbreak detection and response. In 2019, the World Health Organization (WHO) African Regional Office (AFRO) began transitioning from Epi Info, a free, CDC-developed statistical software package with limited capability to integrate with other information systems, affecting reporting timeliness and data use, to District Health Information Software 2 (DHIS2). DHIS2 is a free and open-source software platform for electronic aggregate Integrated Disease Surveillance and Response (IDSR) and case-based surveillance reporting. A national-level reporting system, which provided countries with the option to adopt this new system, was introduced. Regionally, the Epi Info database will be replaced with a DHIS2 regional data platform. This report describes the phased implementation from 2019 to the present. Phase one (2019–2021) involved developing IDSR aggregate and case-based surveillance packages, including pilots in the countries of Mali, Rwanda, and Togo. Phase two (2022) expanded national-level implementation to 27 countries and established the WHO AFRO DHIS2 regional data platform. Phase three (from 2023 to the present) activities have been building local capacity and support for country reporting to the regional platform. By February 2024, eight of 47 AFRO countries had adopted both the aggregate IDSR and case-based surveillance packages, and two had successfully transferred VPD surveillance data to the AFRO regional platform. Challenges included limited human and financial resources, the need to establish data-sharing and governance agreements, technical support for data transfer, and building local capacity to report to the regional platform. Despite these challenges, the transition to DHIS2 will support efficient data transmission to strengthen VPD detection, response, and public health emergencies through improved system integration and interoperability.

## Introduction

Vaccine-preventable disease (VPD) surveillance is critical to public health because it provides data for timely detection of and response to VPD cases and outbreaks. High-quality and timely data are needed to guide program management, tailor public health strategies, and make decisions to achieve program goals (*1*,*2*). In 2023, the World Health Organization’s African Regional Office (WHO AFRO) Universal Health Coverage/Communicable and Noncommunicable Diseases Cluster launched Ending Disease in Africa (ENDISA),* which prioritizes the practice of data-driven precision public health through data integration and advanced analytics to strengthen the availability of high-quality data for decision-making (*3*).

In the WHO African Region,^†^ Epi Info,^§^ a free, CDC-developed statistical software package first released in 1985, has historically been used for aggregate and individual-level (case-based) VPD surveillance data management at the district, provincial, national, and regional levels. However, the Epi Info–based system was limited by delays in data reporting and a lack of ability to integrate with other information systems (*4*). District Health Information Software 2 (DHIS2)^¶^ is a fully customizable open-source health management information system with improved system integration and interoperability features.** DHIS2 is used by approximately 80 low- and middle-income countries worldwide and is used increasingly in the WHO African Region for managing aggregate and individual-level data.

In 2019, a WHO AFRO consultation on integrated VPD surveillance information system management was held to address the limitations of Epi Info and the need for a more timely information system to effectively respond to public health emergencies (*5*). Attendees recommended using DHIS2 as the VPD surveillance regional platform. DHIS2 aggregate Integrated Disease Surveillance and Response (IDSR) and VPD case-based surveillance modules (packages) were developed to replace WHO AFRO’s centralized offline Epi Info VPD information system. Improvements included automatic data synchronization with the regional platform, integration of reporting of multiple diseases into a single database, and increased access to VPD surveillance data at subnational levels. This report describes the phased transition from Epi Info to DHIS2 IDSR and case-based surveillance packages in WHO African Region countries during 2019–2023, and establishment of the WHO AFRO DHIS2 regional data platform.

## Methods

### Development of DHIS2 IDSR and Case-Based Surveillance Packages and Initial Pilots

During the first phase of implementation (2019–2021), development of the DHIS2 IDSR aggregate and case-based surveillance packages included defining user and system requirements; configuring database and user permissions; and configuring organization units, data elements, data entry forms, and user roles. The packages were piloted in three countries: Mali, Rwanda, and Togo.^††^ Countries then selected specific VPDs and data elements for DHIS2 reporting based on national guidelines and in compliance with regional reporting requirements. DHIS2 was developed at the University of Oslo; country-level package implementation status is monitored through the university’s internal tracking tool.

### Expansion of Implementation and Development of the DHIS2 Regional Platform

Phase two (2022) included two activities: expansion of implementation into additional countries and development of the regional platform. First, package implementation was expanded to additional countries using selection criteria similar to those used for the pilot, as well as lessons learned from the pilot. Second, the WHO AFRO DHIS2 regional platform was developed. This platform replaced Epi Info and served as a repository for all reported VPD surveillance aggregate and case-based surveillance data from countries in the region. User and technical requirements were identified by stakeholders.

### Building Capacity and Support for Implementation of the Regional Data Platform

Phase three (from 2023 to the present) focuses on building local capacity and providing technical assistance in the planning, implementation, and evaluation processes to support country-specific reporting to the WHO AFRO DHIS2 regional platform. Activities include training, technical assistance, and ongoing monitoring to ensure successful data integration with the regional platform. During this phase, pilot countries share lessons learned on reporting to the regional platform. This activity was reviewed by CDC, deemed not research, and was conducted consistent with applicable federal and CDC policies.^§§^

## Results

### 2019–2021: Development of DHIS2 IDSR and Case-Based Surveillance Packages and Initial Pilots

DHIS2 IDSR and case-based surveillance packages were developed as options for use at the country level. The DHIS2 IDSR package was used to collect weekly aggregate information on epidemic-prone diseases.^¶¶^ Countries have the flexibility to expand the number of diseases in the packages based on their specific needs and requirements, potentially extending beyond the mandated diseases outlined by International Health Regulations.*** The DHIS2 case-based surveillance package supports individual-level reporting of nine notifiable VPDs^†††^ and is capable of linking clinical, laboratory, case investigation, and outcome data to a single case. Features of the web-based packages are that they can be accessed anywhere with Internet connectivity (enabling near real-time reporting of suspected cases), text or email notifications can automatically be sent to a predefined list of recipients; and data can be pushed to other DHIS2 packages using outbreak thresholds^§§§^ (L Pezzoli, L Noubi Tchoupopnou Royd, WHO, unpublished data, 2023). At the end of this first phase, both packages were partially or fully implemented in the three pilot countries.

### 2022: Expansion of Implementation and Development of the Regional Platform

**FIGURE 1:**
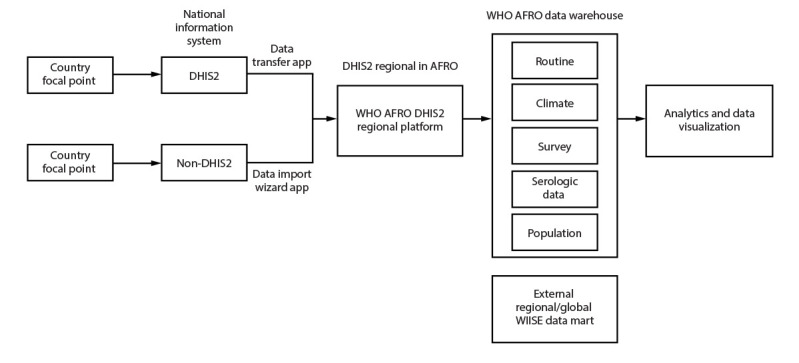
District Health Information Software 2 vaccine-preventable disease surveillance data flow, from country to regional and global levels — World Health Organization African Region* **Source: **Adapted from the WHO AFRO Data Platform vision presentation, WHO AFRO DHIS2 regional workshop, Kigali, Rwanda, March 2023. **Abbreviations: **DHIS2 = District Health Information Software 2; WHO AFRO = World Health Organization African Regional Office; WIISE = World Health Organization Immunization Information System. * The WIISE data mart is a relational database that stores transactional data, facilitating access and organization, and enabling ascertainment of data trends.

The WHO AFRO DHIS2 regional data platform was developed to enhance country-level VPD surveillance reporting to the regional level. VPD surveillance data are reported through an improved integrated and interoperable regional data platform. The platform is a component of the Universal Health Coverage/Communicable and Noncommunicable Diseases Cluster integrated data warehouse that serves as a central data repository to support data analytics and visualization for the region. Data from the warehouse can be shared with external data portals such as the WHO Immunization Information System, which allows users to interact and share immunization data globally (Figure 1) (*6*).

### 2023–Present: Building Local Capacity and Supporting Regional Data Platform Implementation

**FIGURE 2:**
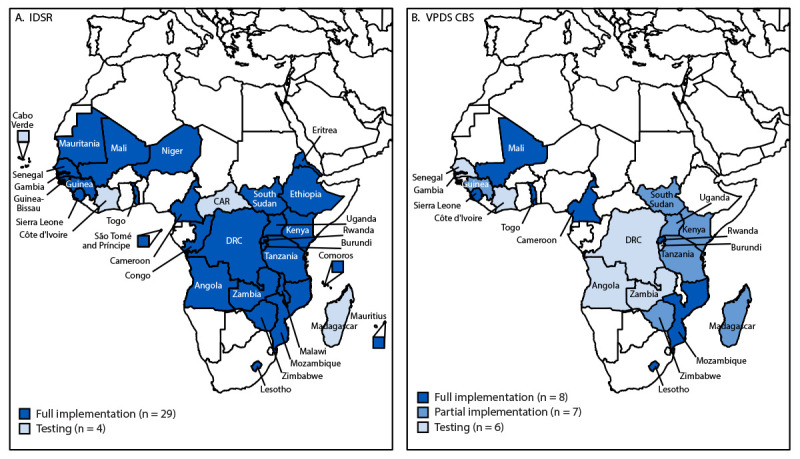
Status of Integrated Disease Surveillance and Response (A) and vaccine-preventable disease case-based surveillance (B) District Health Information Software 2 implementation, by country* — World Health Organization African Region, 2019–2023 **Abbreviations**: CAR = Central African Republic; CBS = case-based surveillance; DRC = Democratic Republic of the Congo; IDSR = Integrated Disease Surveillance and Response; VPDS = vaccine-preventable disease surveillance. * The phase one pilot countries were Mali, Rwanda, and Togo.

As of February 2024, among the 47 WHO African Region member countries, 29 (including the three pilot countries), had fully implemented the IDSR package nationwide, and an additional four countries were in the testing phase (University of Oslo, unpublished data, 2023) (Figure 2). Six countries were testing the case-based surveillance package, seven had partially implemented the package in selected districts, and eight (including the three pilot countries) had fully implemented the package in all districts. In March 2023, seven member countries^¶¶¶^ participated in a WHO AFRO DHIS2 regional onboarding workshop, at which data integration and scale-up implementation plans were developed to connect national data systems to the WHO AFRO DHIS2 regional platform. Participating countries reported that the process of mapping metadata from Epi Info to DHIS2 was time-consuming and that technical support for data transfer was needed to address nonmatching between data elements and organizational units. The countries expressed the need for capacity building to support implementation at both national and subnational levels. Some countries requested formal communication and data-sharing procedures from WHO AFRO to facilitate reporting to the regional platform.

As of February 2024, eight countries had fully adopted both the IDSR and case-based surveillance packages at a national level, and two had successfully transferred aggregate data to the WHO AFRO regional platform using the DHIS2 data transfer app.**** None of the countries using non-DHIS2 data systems had been trained in DHIS2 reporting or had participated in a DHIS2 onboarding workshop; thus, none of these countries had successfully transferred their data using the DHIS2 data import wizard application,^††††^ a tool that supports the transfer of data from a non-DHIS2 information system to a DHIS2 system in various formats^§§§§^ (Figure 1).

## Discussion

To address the need for more efficient data transmission, WHO AFRO, in collaboration with global partners, developed the DHIS2 IDSR and case-based surveillance packages and a regional data platform. The use of the IDSR and case-based surveillance packages will enable direct reporting to the WHO AFRO DHIS2 regional platform.

Despite the progress made to date, regional-level challenges in transitioning from Epi Info to DHIS2 remain. Whereas country-level VPD surveillance data management can leverage partner support by using existing financial and human resources for other funded programs, no similar dedicated resources to support DHIS2 regional VPD surveillance reporting to WHO AFRO currently exist. Financial resources and a workforce capable of customizing and configuring the system are needed (L Pezzoli, L Noubi Tchoupopnou Royd, WHO, unpublished data, 2023); wthout such dedicated resources, transition to the new platform will be delayed, and timely and accurate reporting hindered. In addition, established data-sharing agreements with countries reporting to the regional platform are needed to ensure standardized, timely, secure, and efficient information exchange to provide high-quality data for decision-making.

WHO AFRO is developing operational guidance for DHIS2 regional VPD surveillance reporting to support countries in assessing their readiness and critical considerations for planning and implementation at national and subnational levels. Plans are underway to finalize standard operating procedures for reporting to the WHO AFRO DHIS2 regional platform, as well as formal communications to countries. Developing a well-defined regional transition plan that outlines a clear roadmap, needed resources, and timeline will be vital to ensuring successful DHIS2 implementation for all WHO African Region countries.

### Limitations

The findings in this report are subject to at least two limitations. First, information on the status of country-level implementation on the DHIS2 IDSR and case-based surveillance packages is not routinely updated and relies on unverified self-reports from countries. Second, feedback was provided only by participants from the seven countries who attended the workshop organized by WHO AFRO; feedback from national program managers from other countries was not available, which limits the scope of the findings presented and the applicability of the results to those countries.

### Implications for Public Health Practice

The DHIS2 IDSR and case-based surveillance packages facilitate aggregate and individual-level reporting of epidemic-prone diseases and outbreaks in the WHO African Region. The transition to DHIS2 offers the potential for more efficient information transmission through improved system integration and interoperability, which is crucial for data-driven decision-making and timely detection and response to VPDs and public health emergencies as well as improvement of health in the region.

## Footnotes

*
https://espen.afro.who.int/updates-events/updates/who-afro-launches-groundbreaking-strategy-to-end-diseases-in-africa

† The WHO African Region comprises 47 countries: Angola, Benin, Botswana, Burkina Faso, Burundi, Cabo Verde, Cameroon, Central African Republic, Chad, Comoros, Côte d'Ivoire, Democratic Republic of the Congo, Equatorial Guinea, Eritrea, Eswatini, Ethiopia, Gabon, Ghana, Guinea, Guinea-Bissau, Kenya, Lesotho, Liberia, Madagascar, Malawi, Mali, Mauritania, Mauritius, Mozambique, Namibia, Niger, Nigeria, Rwanda, São Tomé and Príncipe, Republic of the Congo, Senegal, Seychelles, Sierra Leone, South Africa, South Sudan, Tanzania, The Gambia, Togo, Uganda, Zambia, and Zimbabwe.

§ Epi Info is used for aggregate and case-based VPD surveillance data management at the district, provincial, and national levels. This free data management, analysis, and visualization software supports the preparedness, detection, risk assessment, and response to epidemic and pandemic-prone diseases in the WHO African Region. Epi Info uses a Microsoft Access–compatible database for data analysis, mapping, generating Epi Reports, and creating customized application interfaces. https://www.cdc.gov/epiinfo/index.html

¶ DHIS2 is an open-source, web-based software platform for data collection, management, and analysis. DHIS2 is the world’s largest health information management system platform, used by ministries of health in 80 low- and middle-income countries. Approximately 3.2 billion persons (40% of the world’s population) live in countries where DHIS2 is used. With the inclusion of nongovernmental organization–based programs, DHIS2 is in use in approximately 100 countries. https://dhis2.org/in-action/ (Accessed March 26, 2024).

**
https://dhis2.org/overview/

†† Mali, Rwanda, and Togo were selected as pilot countries based on their preparedness and willingness to use the new DHIS2 packages, the maturity of their information systems, and the availability of technical support. These factors made them excellent candidates for testing the IDSR and case-based surveillance packages, enabling a thorough evaluation and refinement before broader implementation.

§§ 45 C.F.R. part 46, 21 C.F.R. part 56; 42 U.S.C. Sect. 241(d); 5 U.S.C. Sect. 552a; 44 U.S.C. Sect. 3501 et seq.

¶¶ Weekly aggregate epidemic-prone diseases include acute flaccid paralysis, acute watery diarrhea, cholera, dengue fever, diarrhea with blood, diphtheria, measles, meningitis, neonatal tetanus, nonneonatal tetanus, pertussis, rabies, rubella, viral hemorrhagic fevers, and yellow fever.

***
https://cdn.who.int/media/docs/default-source/documents/emergencies/case-definitions-ihr-four-diseases7f1ee707-3d13-4581-a1af-d5f44f86423a.pdf?sfvrsn

††† Notifiable VPDs (i.e., congenital rubella syndrome, invasive bacterial vaccine preventable disease, measles and rubella, meningitis, neonatal tetanus, polio [acute flaccid paralysis], rotavirus, rotavirus impact, and yellow fever).

§§§
https://docs.dhis2.org/en/implement/health/disease-surveillance/vpd-case-surveillance/design.html

¶¶¶ Cameroon, Côte d'Ivoire, Mali, Rwanda, South Sudan, Togo, and Uganda.

**** Data transfer application facilitates DHIS2-to-DHIS2 data exchange. This application requires matching, because data might not exactly be aligned in terms of names, codes, or identities.

†††† Supports data transfer in formats such as comma-separated values (CSV). This process requires mapping and matching the information and directly importing the data into DHIS2.

§§§§ Formats such as CSV and Microsoft Excel (xls and xlsx).
